# Exploring Lived Experiences of Men Diagnosed With Prostate Cancer in Oman: A Qualitative Study

**DOI:** 10.1111/hex.70695

**Published:** 2026-05-16

**Authors:** Khalood Al‐Abri, Mazin Hamood Al‐Louyahi, Hassan Said Al‐Samani, Mohammed Juma Al‐Ghanboosi, Khadem Hamed Al‐Junaibi, Nasser Hamood Al‐Adawi, Debby Syahru Romadlon

**Affiliations:** ^1^ Department of Community & Mental Health, College of Nursing Sultan Qaboos University Muscat Oman; ^2^ College of Nursing Sultan Qaboos University Muscat Oman; ^3^ Sultan Qaboos Comprehensive Cancer Care and Research Center Muscat Oman; ^4^ Faculty of Nursing Chulalongkorn University Bangkok Thailand

## Abstract

**Background:**

Life after prostate cancer treatment may extend across decades; however, qualitative evidence describing how men in Oman navigate treatment sequelae, disclosure, family involvement, and religious coping remains limited. This study explored the lived experiences of Omani men who had completed primary therapy for prostate cancer.

**Methods:**

Between September and November 2025, 16 Omani men attending routine follow‐up at a specialised tertiary care centre were invited to take part. Each participant granted a single, audio‐recorded, semi‐structured interview that was transcribed verbatim and analysed by thematic analysis.

**Results:**

Five entangled themes were generated: (1) Silent onset: most men misread early symptoms as ‘normal ageing’ and delayed presentation. (2) Family arbitration: wives, sons and uncles often negotiated the choice between surgery, radiotherapy or androgen‐deprivation therapy. (3) Persistent corporeal shifts: urinary leakage and sexual dysfunction symptoms intruded on prayer routines, market visits and sleep. (4) Emotional oscillation: an initial surge of distress gave way to selective disclosure and gradual recalibration of self. (5) Faith as ballast: daily prayers, Qur'anic recitation and kin obligations supplied a stabilising counter‐weight to uncertainty.

**Conclusion:**

Life after prostate cancer in Oman is shaped by biomedical sequelae, Islamic practice and extended‐family logic. Programmes that integrate relatives into follow‐up consultations and legitimise spiritual coping may reduce unmet psychosocial need more effectively than clinician‐only reviews.

## Patient or Public Contribution

Men living with and beyond prostate cancer participated in this study through interviews. The interview guide was pilot tested with three healthcare providers involved in prostate cancer care and refined based on feedback from the initial participant interviews.

## 1 Introduction

Noncommunicable diseases have lately been identified as major causes of mortality worldwide, with cancer projected to be the leading cause of death and the single most critical barrier to extending life expectancy in every country in the twenty‐first century. According to World Health Organization (WHO) figures from 2015, cancer is the primary or second leading cause of death before the age of 91 in 172 countries [1]. Globally, prostate cancer is the second leading type of cancer among men [2]. Increases in 5‐year survival rates for prostate cancer have been reported in several countries: survival improved by 10–20% from 1995–1999 to 2005–2009 across 22 nations in South America, Asia, and Europe; however, survival rates remain highly variable globally, ranging from < 60% in Bulgaria and Thailand to ≥ 95% in Brazil, Puerto Rico, and the United States [3]. This wide variation may be attributed to disparities in access to early diagnosis and optimal therapy.

This study is framed by an interpretive perspective in which life after prostate cancer is shaped by the interaction between treatment‐related bodily change and the social meanings attached to those changes. Norms of masculinity and stigma influence what can be disclosed, how symptoms are managed in everyday life, and when support is sought, while family relationships and Islamic practice provide resources through which illness is interpreted and negotiated. Health‐system pathways, including diagnostic routes and follow‐up models, also shape what forms of support are available and how needs are voiced. The literature reviewed below is organised around these interlinked clinical and sociocultural processes to provide a coherent rationale for exploring lived experience in Oman.

Clinical pathways shape the post‐treatment trajectory. Localised prostate cancer presents a branching path: androgen‐deprivation therapy, nerve‐sparing prostatectomy, or fractionated external‐beam radiation. Once metastases appears the menu lengthens – systemic hormonal blockade, watchful waiting, or intermittent chemotherapy – yet none of these options is lightweight [4, 5]. Screening and diagnostic policies also shape the point at which men enter these pathways; for example, in the United Kingdom, where no national screening programme exists, symptomatic men – or those with a positive family history – are invited to request a prostate‐specific antigen (PSA) assay and a digital rectal examination [5, 6].

The aftermath of treatment arrives later. Months after the last fraction or the final LHRH injection, sexual function often drifts, urinary continence hesitates, and bowel habits alter their rhythm; fatigue, weight gain, hot flushes and muscle atrophy complete the ledger [4, 5, 7]. Meta‐analysis places post‐treatment depression and anxiety between 15% and 27% – roughly twice the community norms [8]. Identity, meanwhile, feels suddenly porous; men describe themselves as unplugged from previous social circuits, unable to locate the steering wheel they once took for granted [9].

Stigma compounds the damage. Larkin et al. [10] map how internalised shame, self‐selected isolation and downward recalibration of self‐worth erode global quality‐of‐life scores. Richardson et al. [11] counter that participants who re‐cast cancer as a time‐limited challenge – not an irreversible deficit – deploy more active coping and report less intrusive distress.

Such recasting is continuous. Illness beliefs are not archaeological artefacts; they are revised incrementally as bodies age, as sexual function declines, and as the cultural syntax of masculinity is reformulated. Prostate cancer merely renders the revision visible. In a US phenomenological study, participants kept returning to the question ‘who am I now?’ and answered by pointing to pads, leaks and the absence of morning tumescence [12]. An Irish cohort refused to frame life after treatment as a discrete stage; recovery, they insisted, behaves like weather – calm, storm, calm – rather than a finish line [13].

Across thirty‐one qualitative investigations, three motifs recur: stigma, selective disclosure, and the apprehension of being judged ‘less of a man’ [14]. The emotional valence of these motifs, however, is geographical: whispered in Riyadh clinics, articulated in Dublin pubs, disparaged in São Paulo gyms. Coping is negotiated relationally; when patient and partner narratives converge, mutual support follows – when they diverge, quiet resentment is forecast [15]. Rivas et al. [16] demonstrated that the same symptom – urinary incontinence, for example – can be interpreted as divine trial, honour blemish, or family secret, depending on latitude and liturgical calendar.

Qualitative research from Arab contexts has expanded in recent years, including interpretive work on prostate cancer experiences; however, studies focused specifically on everyday life after treatment remain uneven across countries, and evidence from Oman is limited. There are no structured post‐treatment programmes, no sanctioned lexicon for discussing side‐effects, and only fragmentary referral pathways for late‐onset sequalae. This study explored the lived experiences of Omani men living with and beyond prostate cancer, with attention to psychological challenges, coping practices, and support systems across the illness trajectory and post‐treatment life.

## 2 Methods

### 2.1 Research Design and Study Setting

This study was guided by hermeneutic phenomenology informed by Heidegger and employed in‐depth individual interviews to explore the lived experiences of men living with and beyond prostate cancer in Oman. Because post‐treatment narratives may involve private, culturally sensitive issues, including embodied changes and shifts in identity, the interview approach was paced and handled with care to allow participants to pause, decline to elaborate, or redirect discussion when needed. Methodological congruence was maintained by keeping the design, sampling, and interviewing strategy aligned with a phenomenological aim, to elicit meaning as described by participants rather than to quantify or compare experiences. The study took place at a specialised tertiary care centre where participants attended routine follow‐up appointments between September 2025 and November 2025. This site was selected because it is a specialised cancer hospital serving men from different governorates and socio‐economic backgrounds across Oman.

### 2.2 Sampling Techniques and Sample Size

The study employed the purposive sampling that allows researchers to deliberately select samples according to certain characteristics, which in turn helps study the lived experiences of men living with and beyond prostate cancer. The study included Omani males diagnosed with prostate cancer who had completed primary treatment, who were 18 years old or older, understand the Arabic language enough to respond to the questions and can provide informed consent agreed to participate in the study.

Non‐Omani or who were less than 18 year, or Omani males diagnosed with cancers other than prostate cancer, who were unable to understand the Arabic language and unwilling to participate in the study were excluded.

#### 2.2.1 Data Collection Procedures

Before conducting the study, the ethical approval was obtained from relevant institutional ethics committees (CON/GP/2024/25 and SQCCCRC‐IRB‐EC‐2025‐74). Then the assigned nurse, who is a part of this research and works in the specialised tertiary care centre's unit of prostate cancer patients, assisted the researcher in approaching the patients, explaining the procedure, signing the consent form, and providing a private room in the unit to conduct the interviews. Before commencing interviews, the semi‐structured interview guide was pilot tested with three healthcare providers who work directly with men living with and beyond prostate cancer at the centre. They were to evaluate the clarity, clinical relevance, and cultural sensitivity of the interview questions. Some examples of changes: the phrasing ‘psychological and social challenges’ was changed to ‘emotional or mental difficulties’ so that people could ensure participants from the local context could easily understand the question. Furthermore, the question on coping mechanisms was changed to ‘Can you describe specific methods you use to manage stress or anxiety related to your condition?’ to make it more practical and understandable. Following this preliminary modification, the interview guide was distributed to the first participants diagnosed with prostate cancer. During these initial interviews, we closely monitored participants' reactions, comfort levels, and input on question phrasing and sensitivity. Minor changes were made to improve clarity and cultural appropriateness where needed. These first patient interviews were fully included in the final analysis because no significant structural adjustments were necessary to the interview guide.

A few changes were made to the guide based on feedback received from the participants. All interviews took place at the selected specialised tertiary care centre, through face to face and open questions interviews that were selected based on the criteria and goals of the research to guarantee uniformity throughout the interviews (See appendix A). Twenty participants were invited; four were excluded due to their relatives seeking to participate in the interviews. Sixteen participants were interviewed, achieving data saturation as no additional information emerged. Saturation was assessed iteratively through probing to enhance responses, reflexive journaling to identify emerging patterns, and monitoring the recurrence of codes/themes to identify the point at which no new data appeared [17]. The participants had given their consent if they were willing and wish to proceed with the interview and were informed about their right to withdraw from the study at any time without any penalty. The interviews were recorded and securely saved in a password‐protected file, granting access solely to the study team members. The interviews lasted between 25 and 40 min and were held in a private setting. All researchers who conducted the interviews to take notes, transcribed recordings verbatim, and verified each transcript against the audio.

#### 2.2.2 Data Analysis

All interviews were conducted in Arabic, transcribed verbatim, and checked against the audio‐recordings before analysis. Data were analysed using reflexive thematic analysis as outlined by Braun and Clarke [18], consistent with the study's hermeneutic phenomenological orientation informed by Heidegger and an interpretive approach to meaning‐making. In this study, reflexive thematic analysis was used as a practical analytic tool to organise and interpret participants' accounts, while the overall analysis remained guided by a phenomenological focus on meaning and lived experience. The emphasis was on interpreting participants' experiences rather than simply categorising data, which aligns with the aims of hermeneutic phenomenology. Thus, reflexive thematic analysis supported by providing a clear and systematic way to develop themes grounded in participants' lived meanings.

Analysis was iterative and context‐sensitive: each transcript was read in full and re‐read during coding and theme refinement, with the team moving between whole accounts and meaning‐dense excerpts in a whole‐to‐part manner consistent with the hermeneutic circle. Initial codes were generated through close, line‐by‐line engagement with the data, supported by analytic memos and reflexive notes to document developing interpretations. The PI (KA) and co‐investigators (MA, HA, MA and KA) discussed and refined codes and candidate themes through regular meetings, with themes reviewed for internal coherence and clear distinctions, and continuously checked back against the dataset and the study aims. The report was produced by integrating illustrative participant quotations to support themes and present a coherent account of participants' meaning‐making.

#### 2.2.3 Rigour and Trustworthiness

Rigour was addressed through methodological congruence, reflexivity, dependability, and confirmability, alongside established trustworthiness practices. Methodological congruence was supported by aligning the research aim (lived experience), the use of in‐depth interviews in a private setting, purposive recruitment of participants able to narrate experiences in Arabic, and an analytic approach that retained context and meaning within each account. Reflexivity was treated as ongoing rather than incidental: the PI kept a reflexive journal from September to November 2025, documenting interpretive assumptions, analytic hunches, and methodological decisions, and these reflections were used to check how prior expectations could shape theme development [19]. Dependability was strengthened through analyst triangulation and a transparent analytic process, with multiple co‐investigators coding the same transcripts independently and reconciling differences through regular meetings with the PI; agreement on the refined coding scheme supported stability in how codes and themes were applied across the dataset. Confirmability was strengthened by maintaining an auditable chain from raw data to findings. Audio recordings were transcribed verbatim and verified, marginal notes were converted into analytic memos, and transcripts were revisited after time gaps as part of deliberate slow coding to test whether interpretations remained supported when early impressions had faded. Member checking further supported confirmability; consolidated themes and exemplar quotations were returned to participants in Arabic or English (as preferred) and amended where meaning had drifted [20]. The final manuscript was prepared with reference to the SRQR checklist, with participant counts reported at both interview and member‐check stages to indicate attrition [21].

## 3 Results

The study included a total of 16 men diagnosed with prostate cancer participated in this study. The participants ranged in age from 58 to 86 years. Most were diagnosed with Stage T2 or T3 cancer. Treatment modalities varied, with participants undergoing radiation therapy, hormonal therapy, prostatectomy, or a combination of these (Table 1).

**Table 1:** Sociodemographic characteristics of the study participants (*N* = 16).

N.o	Age	Stage by TNM	Marital status	Living arrangement	Educational level	Employment status	Date of diagnosis	Treatment modality
1	75	T3N2M0	Married	Lives with family	No formal education	Retired	2022	Prostatectomy + Radiation
2	75	T2N0M0	Married	Lives with family	Primary school	Retired	2018	Radiation + Hormonal
3	69	T2N0M0	Married	Lives with family	Primary school	Employed	2023	Radiation
4	58	T2N1M0	Not Married	Lives alone	Diploma	Employed	2022	Radiation + Hormonal
5	60	T3N0M1	Married	Lives with family	Primary school	Employed	2020	Radiation + Hormonal
6	68	T3N0M0	Married	Lives with family	Primary school	Employed	2019	Radiation + Hormonal
7	69	T2N0M0	Married	Lives with family	Diploma	Retired	2018	Radiation
8	61	T3N0M0	Married	Lives with family	Masters	Employed	2024	Radiation + Hormonal
9	68	T2N1M0	Married	Lives with family	No formal education	Employed	2022	Hormonal
10	65	T3N2M0	Married	Lives with family	Bachelors	Employed	2024	Radiation + Hormonal + Prostatectomy
11	73	T3N0M0	Married	Lives with family	No formal education	Retired	2023	Radiation + Hormonal
12	67	T3N1M1	Married	Lives with family	Secondary school	Employed	2019	Radiation + Hormonal
13	81	T2N1M0	Married	Lives with family	No formal education	Retired	2022	Radiation + Hormonal
14	86	T2N1M1	Married	Lives with family	No formal education	Retired	2021	Prostatectomy + Radiation
15	72	T2N1M0	Married	Lives with family	Secondary school	Retired	2019	Radiation + Hormonal
16	72	T2N0M0	Married	Lives with family	No formal education	Retired	2020	Radiation + Hormonal

## 4 Themes

The analysis of semi‐structured interviews yielded five recurring patterns that organise the lived experience of men living with and beyond prostate cancer in Oman: (1) silent onset, (2) family arbitration, (3) persistent corporeal shifts, (4) emotional oscillation, and (5) faith as ballast. Each pattern comprises two or more sub‐currents that capture distinct yet overlapping dimensions of the participants' trajectory.

Theme 1 Silent Onset: early experiences of prostate cancer

This pattern encompasses the pre‐diagnostic phase and the immediate aftermath of receiving a diagnosis. Two sub‐currents were identified: the route by which men recognised that something was wrong, and the affective responses that followed clinical confirmation.

### 4.1 Knowledge and Pathways to Diagnosis

Most participants entered the illness narrative with minimal prior knowledge of prostate cancer. Lower‐urinary‐tract symptoms – hesitancy, nocturia, weak stream – were routinely attributed to benign processes such as ageing, diabetes, or uncomplicated infection, thereby generating presentation delays ranging from four to eighteen months. Clinical entry was typically precipitated by either an acute event (urinary retention, visible haematuria) or the intervention of a proactive female relative who scheduled the appointment. Prostate‐specific antigen testing was uniformly unfamiliar; participants could not decode the numeric results, which amplified the shock of diagnosis. P2 admitted, ‘*Honestly, I had no knowledge about anything. I accompanied my brother for checkups and learned about the screening*.’ P5 normalised his symptoms, ‘*I had inflammation of the prostate for a long time, it came and went, and I never thought it was serious*.’ P13 temporalised the drift, ‘*Difficulty in urination… I didn't know exactly what the problem was. Started around 1 year and a bit… maybe July*.’

The diagnostic pathway itself split three ways. The first group presented only after severe retention or pain. P16 recalled, ‘*The urine wouldn't come out… very weak. There was narrowing and blockage. I visited several hospitals… later they suspected a malignant tumo*r.’ P7 described a painful swelling, ‘*At first, the symptoms were a large swelling in that area, with pain. It went away once, then returned a month later*.’

The second subgroup discovered cancer incidentally during routine blood work. P12 explained, ‘*I discovered it by coincidence… I wasn't doing a checkup; the nurse said something in the blood looked off*.’

The third, smallest cluster had a positive family history and screened early. P1 noted, ‘*I was worried because this illness exists in our family, so I checked early*.’

### 4.2 Initial Emotional Reactions

Receiving the verdict triggered heterogeneous affect. Some men described instantaneous shock, death‐related dread and denial, ‘*Anyone at an older age gets scared… I was terrified*’ (P7). Others reported the same sentence yet experienced no surge; instead, they described calm, almost rapid acceptance anchored in religious attribution: ‘*When I discovered I had it, I accepted it. God brings the illness for a reason*’ (P1). P8 went further stating, ‘*I received the news with happiness. I didn't feel pessimistic… I accepted the diagnosis with comfort, faith, and certainty in God's will*.’

Theme 2 Family Arbitration: navigating diagnosis and treatment decisions

Two organising sub‐themes emerged here: first, how participants understood treatment options and appraised risk through family consultation; second, the fragmented referral trajectories that separated decision from implementation.

### 4.3 Understanding Treatment Options, Risk Perception and Role of Family

At diagnosis, clinicians outlined three standard modalities: radical prostatectomy, external‐beam radiation, or androgen‐deprivation injection. Risk appraisal, however, was refracted through personal priorities and family counsel; a notable plurality favoured radiation precisely because it avoided theatre admission and chemotherapy's visible stigmata. P12 explained: ‘*They offered radiation or surgery. I chose radiation… I believe it's better than surgery because surgery has many steps and hospital stays*.’ P14 echoed: ‘*I chose radiation because it had fewer side effects*.’ Family members – wives, sons, uncles – routinely mediated these deliberations, translating jargon and weighing oncological benefits against perceived threats to masculinity and independence.

These decisions were never atomised; they unfolded inside dense kin networks. Prior family experience acted as a veto or a green light: ‘*I consulted my family; they told me surgery is better*’ (P10). One man traced his radiation preference directly to paternal memory, ‘*In the beginning, the main suggestion was radiation therapy. We said let's go with radiation because my father had taken chemotherapy before, and I developed a kind of phobia from it – like the hair loss and all of that*’ (P7).

### 4.4 Complex Treatment Pathways and Referrals

The route from diagnosis to first treatment fraction was rarely linear. Participants shuttled between local polyclinics, regional hospitals and the tertiary site to confirm histopathology. A subset sought second opinions abroad, particularly in India, either to validate local findings or to access robotic surgical capacity unavailable in Oman at that time. ‘*I travelled to India to confirm the tests from Oman Hospital. They matched exactly*’ (P8). ‘*In India they told me they do the surgery using robot‐assisted technique… I travelled to India and did the robotic surgery there*’ (P16).

Theme 3 Persistent Corporeal Shifts: physical impact and daily life changes

This theme catalogues the somatic residues of treatment that mandated daily recalibration. The findings were categorised into two sub‐themes, urinary challenges and daily life adaptations as well as sexual health and function.

### 4.5 Urinary Challenges and Daily Life Adaptations

Urinary dysfunction – urgency, incontinence, post‐void dribbling – dominated participant narratives. Following surgery or radiation, many described erratic bladder control that compromised not only personal hygiene but, more consequentially, ritual purity. The five daily prayers require ablution; minor leakage voided that state. P15 explained: ‘*I still have leakage even after bathing… after a while it comes out. When I feel the urge I cannot hold it; I must rush to the bathroom*.’ P14 articulated a double bind: ‘*I cannot be in a state of ablution, on my way to pray, and then find wetness on my clothes. I also cannot tolerate it when I have guests at my house or when I am a guest at someone else's home. This is something I cannot bear*.’ Despite such encumbrances, participants devised workarounds – meticulous bathroom mapping, fluid restriction before outings – and preserved exercise and social schedules. P4 insisted: ‘*My life is the same – I exercise, travel, and socialise as usual*.’

### 4.6 Sexual Health and Function

Sexual dysfunction was reported yet minimally detailed. Participants treated the topic as culturally delicate, offering general statements and evading self‐presentation as diminished. Most men on hormonal therapy or radiation acknowledged decreased libido and erectile impairment; a minority, however, framed such changes as normative ageing, thereby deflecting stigma. P5 noted: ‘*From the start of hormone therapy, I had reduced sexual desire and erectile weakness*.’ P3 stated: ‘*The prostate is weak now. Things are not like before (when young). You know, in intercourse*.’

Theme 4 Emotional Oscillation: psychosocial responses and adjustment

This theme encompasses participants' internal emotional trajectories and their social navigation post‐diagnosis. The analysis distilled three interrelated sub‐themes: emotional distress and shock, the cultivation of acceptance and resilience, and the strategic management of stigma through selective disclosure.

### 4.7 Emotional Distress and Shock

A prostate‐cancer diagnosis typically precipitated acute emotional disequilibrium. The term itself – unanticipated and freighted with mortality – amplified worry, sadness, and anxiety that rippled into the immediate family. P3 recalled: ‘*On the first day when the doctor told us, I was very affected and my children too*.’ P10 described concurrent depression and fleeting anxiety, noting that ‘*My feelings weren't good, I had depression and anxiety… Acceptance made things easier*.’ For P7, the first 3 months constituted a period of sustained alarm: ‘*exhausting, really exhausting… I was just afraid it would continue*.’

### 4.8 Acceptance and Emotional Resilience

Most participants, in contrast, traced a path toward emotional stabilisation. Many normalised the illness as a manageable ‘part of life,’ refusing to grant it centrality in their identity. P14 mentioned: ‘*I stayed happy and didn't let negative thoughts affect me*.’ P12 reported a deliberate cognitive strategy: ‘*Psychologically, as long as you don't think of the disease, there is no problem*.’ P6 historicised his mortality, stating: ‘*Life is still the same… People eventually passed away; our grandparents passed on, and we will too… Once [a person] accepts things, they find relief*.’

### 4.9 Navigating Stigma and Selective Disclosure

Whom to tell, and whom to omit, became a delicate negotiation. Fear of gossip, pity or misdiagnosis (several men noted neighbours conflating prostate cancer with HIV/AIDS) motivated selective silence. P11 withheld information from extended family: ‘*I didn't tell anyone, even the family… only my children knew. If you tell many people, everyone will give you different advice and you get confused*.’ P7 explained, ‘*It's better to keep things brief… if you tell everyone, they will spread the news… In the neighbourhood, people will start saying things like: ‘They have AIDS' or other illnesses*.’

Theme 5 Faith as Ballast: coping strategies and support systems

This theme catalogues the external scaffolding and internal armoury participants mobilised. Two pillars emerged: (1) religious sense‐making and (2) immediate‐family solidarity.

### 4.10 Religious Sense‐Making

Faith functioned as the most consistently invoked adaptive repertoire. Participants reframed the illness not as stochastic misfortune but as divine test (ibtila') or pre‐ordained decree, a re‐labelling that attenuated death‐anxiety and furnished existential ballast. Prayer, Quranic recitation and personal supplication were cited as daily emotional regulation practices. P7 asserted, ‘*99% of my strength came from being Muslim… Muslims read Quran and keep calm. Everything is God's decree*.’ P8 described a seamless spiritual integration: ‘*I accepted the diagnosis with comfort, faith, and certainty in God's will*.’ P5 normalised suffering theologically, ‘*I considered it a test from God; religion encourages patience*.’ For P16, belief dissolved stress, ‘*Believing in Allah and accepting fate reduces the stress*.’ P1 reported increased devotional intensity, ‘*I don't fear death… I started praying more, reading, and praying late at night*.’

### 4.11 Immediate‐Family Solidarity

Wives and children provided logistical and emotional scaffolding. Offspring scheduled appointments, chaperoned radiation fractions and accompanied fathers to India for robotic surgery, acts described as ‘essential’ for adherence and morale. P1 recalled, ‘*My children took turns accompanying me. My whole family supported me*.’ P16 noted transnational coordination: ‘*My siblings helped me, even in travelling for treatment… My family stayed with me during treatment abroad*.’ P8 summarised simply saying, ‘*My wife and children supported me fully*.’

Yet a deliberate boundary was traced around the nuclear unit. Extended relatives were routinely excluded to avoid burden or gossip, reinforcing a compact, trusted circle. As P7 phrased it: ‘*Support should come from inside the house. Outsiders only cause problems*.’

## 5 Discussion

This qualitative study mapped the lived arc of life after prostate cancer among Omani men, tracing early symptom attribution, treatment deliberation, somatic sequelae and adaptive coping. The resulting portrait, though anchored in Gulf cultural specifics, shares some features with international qualitative findings, while also reflecting Oman‐specific meanings and care pathways [22, 23, 24, 25]. Although the account is rooted in Oman, its implications are not confined to one health system. For clinicians and researchers working with Arab and Muslim men, including those in migrant or diaspora communities, the findings underline how treatment choices and later adjustment are often co‐produced with family and anchored in everyday religious practice, shaping what psychosocial support needs to look like in routine follow‐up.

Life after treatment was described as an ongoing negotiation rather than a discrete phase, shaped by the interaction of embodied change, social judgement, and the realities of navigating follow‐up care [23]. While prior qualitative work has described fragmented identity, contested masculinity, and privately managed distress after diagnosis [24], the present findings suggest that everyday adjustment in Oman is additionally mediated by culturally salient meanings attached to urinary and sexual symptoms, including concerns about shame and social exposure [22]. Participants' accounts also showed micro‐adjustments and cognitive reframing aimed at preserving normality, but these strategies appeared to function not only as individual coping, but also as socially protective practices that maintained dignity and family privacy [25]. Taken together, the data point to the value of follow‐up care that addresses function and meaning together, provides confidential routes for discussing sensitive concerns, and reduces discontinuity that can intensify uncertainty [23].

Our first theme, that early urinary symptoms were normalised as benign ageing, deferring presentation, mirrors accounts from Canada and Ireland [24, 26]. In the present sample, most participants described limited prior knowledge of prostate cancer and framed lower urinary tract symptoms as expected or attributed to familiar conditions, which was associated with delays in seeking care that extended from several months to well over a year. Presentation was often triggered only when symptoms escalated, or when a female relative intervened to arrange clinical review, and unfamiliarity with prostate‐specific antigen testing appeared to intensify the shock at confirmation. Hanly et al. [26] described a post‐diagnosis ‘new normal’ in which men struggled to integrate altered urinary and sexual function into self‐concept, that struggle may have earlier origins, beginning before diagnosis when symptom reinterpretation first postpones help‐seeking. This pattern, bodily change interpreted as acceptable rather than alarming, appears across continents and contributes to later‐stage detection and heightened psychological burden at the moment of confirmation [23, 25]. Evidence from longer‐term follow‐up studies further suggests that delayed diagnosis and ongoing functional changes can have lasting psychosocial effects, underscoring the need for improved community awareness of prostate cancer symptoms and post‐treatment support needs [22, 25].

The second theme, treatment decision‐making and navigation through the health system, showed that treatment choices were not made as purely individual or biomedical decisions. Participants interpreted surgery, radiation, and hormonal therapy through family advice, prior illness narratives, and locally circulating perceptions of burden, side effects, and masculinity. In this sense, family involvement did more than provide support, it functioned as an interpretive filter through which treatment risks became understandable and acceptable. Reliance on lay networks and prior family illness stories has been described in other qualitative work [24, 27, 28], but in the present study it also appeared to compensate for fragmented referral pathways and uncertainty across care settings.

Physical and functional consequences of treatment were central in participants' accounts, particularly persistent urinary difficulties and changes related to sexual function, and these issues are known to be linked to reduced quality of life and emotional strain [22, 24, 25]. In this cohort, however, urinary symptoms appeared to carry additional social and religious weight because of their potential to disrupt daily routines and participation in communal life, which shaped coping and help‐seeking. Sexual changes were acknowledged but often discussed indirectly, and several participants reframed them as normative ageing, which may reduce perceived stigma while simultaneously narrowing opportunities to access support [22]. These findings support the need for anticipatory counselling and accessible rehabilitation pathways within routine follow‐up, including continence support and culturally safe sexual health counselling [25, 29]. Longitudinal and follow‐up studies repeatedly associate urinary, sexual, and bowel dysfunction with reduced quality of life and emotional strain, underscoring the importance of anticipatory counselling and access to rehabilitative services – continence training, sexual health counselling, and hormonal symptom management [22, 25, 29].

The fourth theme, emotional oscillation, captured participants' psychosocial responses and adjustment following diagnosis. This theme comprised three related subthemes: initial emotional distress and shock, movement toward acceptance and resilience, and the management of stigma through selective disclosure. Within this framework, selective disclosure emerged as a prominent social strategy. The current study showed that several men limited disclosure outside their immediate family due to fears of gossip, community judgement, or misinterpretation, which are behavioural patterns that reduce social openness and increase emotional isolation. It also noted that participants avoided open discussion of post‐treatment sexual function, a reticence documented across Muslim and Middle Eastern Arab contexts ([30, 31]; Bekele et al., 2025). In the present study, sexual dysfunction was often acknowledged in general terms but minimally elaborated, and several men deflected potential stigma by framing changes as ‘normative ageing’. This pattern can be interpreted as a culturally situated strategy to protect masculinity and marital privacy and to avoid social exposure, but it may also reduce opportunities for clinical assessment and supportive care when discussion relies on spontaneous disclosure [22]. These findings suggest that sexual health concerns should be approached proactively and privately in follow‐up, using culturally sensitive, normalising language and clear permission to discuss symptoms.

A counterpoint to these barriers, faith supplied a durable scaffold. Men routinely reinterpreted malignancy as divine test and prayer as emotional ballast. Parallel studies among Arab Muslim oncology patients reported identical sense‐making; Islamic tenets facilitate acceptance, kindle hope and consolidate kin support [32]. Systematic reviews of coping in Muslim cohorts confirm that religious belief is deployed to render uncertainty bearable and to sustain psychological buoyancy [33]. Quantitative work further demonstrated positive correlation between religiosity and well‐being in Muslim cancer populations [34].

Finally, the fifth theme illuminates the architecture of support as an active coping arrangement rather than a passive backdrop. Support was concentrated within the immediate family, with wives and children providing practical coordination and emotional containment, while disclosure to extended relatives was often restricted to reduce gossip, pity, or misinterpretation. This selective disclosure appeared to function as a protective strategy that preserved control and family privacy, while keeping essential care work within trusted relationships. Although the centrality of family involvement aligns with prior qualitative accounts of role renegotiation after treatment [25], the present findings highlight the boundary‐setting work that accompanied family support and its implications for designing family‐inclusive, confidentiality‐sensitive psychosocial care.

### 5.1 Strengths and Limitations

This study possesses several strengths. First, it addresses a regional evidence gap; Western templates cannot be grafted onto Omani kin structures or Islamic sense‐making. Second, semi‐structured interviews elicited narratives thick enough to trace interactions among bodily symptoms, psychosocial strain, cultural expectation and spiritual coping. Third, the sample spanned ages 58–86, included surgery, radiation and hormonal cohorts, and captured men one to 5 years post‐treatment, thereby sampling multiple positions along the illness trajectory.

Limitations are also acknowledged. Recruitment from a single tertiary centre curtails transferability to peripheral governorates, private clinics or hospitals with different referral logics. Reliance on self‐reported narratives, moreover, invite recall distortion, social‐desirability bias, and selective silence – especially acute in a cultural milieu where masculinity, sexual function and emotional vulnerability are tightly policed. Future work should interview spouses to capture dyadic adjustments and deploy longitudinal designs to track how meanings of living with and beyond prostate cancer drift across years.

### 5.2 Clinical Implication

The findings of this study have important clinical implications for strengthening living‐with‐and‐beyond‐prostate‐cancer care within Oman's healthcare system. Four imperatives emerge. First, spirituality must be embedded – not appended – into post‐treatment care. Oncology nurses and uro‐oncologists should routinely enquire about religious framing, invite prayer schedules into appointment timing and recognise Quranic recitation as a legitimate adjunct to emotional regulation for Muslims. Evidence confirms that spiritual well‐being predicts quality‐of‐life trajectories and should anchor any culturally responsive model [35, 36]. Second, delayed help‐seeking driven by symptom normalisation signals a need for targeted, nurse‐led community education that re‐frames lower‐urinary‐tract symptoms as warranting investigation, not resignation. Such outreach aligns with contemporary post‐treatment care frameworks emphasising health promotion and shared management across the care continuum [37]. Third, the documented stigma, anxiety and selective disclosure demand routine psychosocial screening and explicit referral pathways into counselling or peer‐support. Unmet psychological needs are known to fester when left implicit [35, 36]. Fourth, families function as de facto decision‐makers; clinicians must therefore enrol wives and adult children as recognised partners in treatment selection and long‐term monitoring, consistent with shared‐care models that credit kin networks with driving engagement [35]. Finally, the gaps illuminated – physical rehabilitation, sexual health counselling, fatigue clinics, urinary‐function programmes – require multidisciplinary, nurse‐led or virtual post‐treatment clinics that improve reach and acceptability [37]. Policymakers should standardise diagnostic algorithms to curtail the costly, anxiety‐provoking pilgrimage for second opinions abroad. Collectively, these recommendations argue for a holistic, patient‐and‐family‐centred, culturally consonant model of living with and beyond prostate cancer in Oman.

## 6 Conclusion

This investigation illuminates the lived texture of life after prostate cancer in Oman, where physical sequalae, psychosocial recalibration, cultural prescription and spiritual sense‐making entwine. Participants catalogued marked treatment‐related morbidity, affective distress and disclosure practices filtered through societal norms and stigma dread. Counterbalancing these burdens, faith, kin solidarity and individual resilience functioned as armature – enabling navigation of uncertainty and preservation of hope across the illness trajectory. Such culturally embedded coping underscores the imperative to architect post‐treatment care models congruent with Omani values, belief systems and kin architectures. By weaving cultural sensitivity, spiritual scaffolding and comprehensive post‐treatment planning directly into oncology workflows, healthcare providers can measurably enhance quality of life and long‐term well‐being for men living with and beyond prostate cancer in Oman.

## Author Contributions

**Khalood Al‐Abri:** conceptualisation, methodology, supervision, data curation, project administration, visualisation, writing – review and editing, writing – original draft, validation, investigation. **Mazin Hamood Al‐Louyahi:** investigation, writing – review and editing, writing – original draft, formal analysis, data curation, conceptualisation. **Hassan Said Al‐Samani:** investigation, writing – original draft, writing – review and editing, formal analysis, data curation. **Mohammed Juma Al‐Ghanboosi:** investigation, data curation, formal analysis, writing – original draft, writing – review and editing. **Khadem Hamed Al‐Junaibi:** investigation, writing – original draft, writing – review and editing, data curation, formal analysis. **Nasser Hamood Al‐Adawi:** writing – review and editing, investigation, data curation, supervision. **Debby Syahru Romadlon:** writing – review and editing.

## Funding

The authors have nothing to report.

## Ethics Statement

The ethical approval was obtained from relevant institutional ethics committees (CON/GP/2024/25 and SQCCCRC‐IRB‐EC‐2025‐74). Prior to any data collection, the research information sheet along with an informed consent form was sent to participants, and then consent was obtained. All the collected data collection forms were kept in a locked cupboard in the office of the PI to maintain confidentiality and access only for the research team.

## Conflicts of Interest

The authors declare no conflicts of interest.

## Supporting information

Supporting File

## Data Availability

Data and analysis for this study is available upon request.
