# Supplementary material for: Exploring Lived Experiences of Men Diagnosed With Prostate Cancer in Oman: A Qualitative Study
Source: Health Expect. 2026 May 16;29(3):e70695. doi: 10.1111/hex.70695 (PMC13179749; doi:10.1111/hex.70695)
Supplement: Supplementary file 1 — Supporting File [file HEX-29-e70695-s001.docx]

**Appendix A: Interview Guide**

**Introduction**

Thank you for agreeing to participate in this study. The purpose of this interview is to explore your experiences as a prostate cancer survivor in Oman. Your responses will be kept confidential and used only for research purposes. You may choose not to answer any question and may stop the interview at any time. With your permission, this interview will be recorded to ensure accuracy.

**Opening Questions**

1. Please describe your experience with prostate cancer.
2. What treatment options were available to you, and why did you choose your specific treatment?
3. What lifestyle changes did you make following your treatment?

**Main Interview Questions**

**1. Psychological and Social Challenges**

1. Can you describe any emotional or mental difficulties you experienced?
2. How did your diagnosis affect your relationships with family and friends?
3. Have you experienced any social stigma or changes in your social roles since your diagnosis?

**2. Coping Mechanisms**

1. What coping strategies have you found helpful in managing the challenges associated with prostate cancer?
2. Can you describe specific methods you use to manage stress or anxiety related to your condition?
3. Have you sought professional psychological support or counseling?
4. How do you manage physical symptoms or treatment side effects?

**3. Support Systems**

1. What support systems are available to you in Oman?
2. How effective have these support systems been?
3. Have you participated in any support groups or cancer survivor networks?
4. How has the healthcare system supported you throughout your journey?
5. Are there any community resources or organizations that you found particularly helpful?

**4. Family and Social Support**

1. How has your family and social network contributed to your journey with prostate cancer?
2. In what ways has your family been involved in your care and recovery?
3. Can you describe any specific support or encouragement you received from friends or community members?
4. Have there been any challenges in your relationships due to your cancer diagnosis?

**5. Spirituality and Faith**

1. What role do spirituality and faith play in your coping process?
2. How have your religious or spiritual beliefs influenced your perspective on your illness and recovery?
3. Are there any religious practices or spiritual activities that helped you cope?
4. How did your involvement in your religious community support you during your illness?

**Closing the Interview**

1. Is there anything else you would like to share about your experience with prostate cancer that we have not discussed?
2. Do you have any additional comments or reflections that you feel are important?

**Closing Statement**

Thank you very much for sharing your experiences and insights. Your participation is greatly appreciated and will contribute valuable knowledge to this study. All information shared will remain confidential and will be used solely for research purposes.
